# Knowledge on the transmission, prevention and treatment of malaria among two endemic populations of Bangladesh and their health-seeking behaviour

**DOI:** 10.1186/1475-2875-8-173

**Published:** 2009-07-29

**Authors:** Syed Masud Ahmed, Rashidul Haque, Ubydul Haque, Awlad Hossain

**Affiliations:** 1 BRAC Research and Evaluation Division, BRAC Centre, 75 Mohakhali, Dhaka-1212 Bangladesh; 2 ICDDR, B. GPO Box 128, Dhaka-1000, Mohakhali, Dhaka-1212, Bangladesh

## Abstract

**Background:**

Data on sociological and behavioural aspects of malaria, which is essential for an evidence-based design of prevention and control programmes, is lacking in Bangladesh. This paper attempts to fill this knowledge gap by using data from a population-based prevalence survey conducted during July to November 2007, in 13 endemic districts of Bangladesh.

**Methods:**

A two-stage cluster sampling technique was used to select study respondents randomly from 30 *mauzas *in each district for the socio-behavioural inquiry (n = 9,750). A pre-tested, semi-structured questionnaire was used to collect data in face-to-face interview by trained interviewers, after obtaining informed consent.

**Results:**

The overall malaria prevalence rate in the 13 endemic districts was found to be 3.1% by the Rapid Diagnostic Test 'FalciVax' (*P. falciparum *2.73%, *P. vivax *0.16% and mixed infection 0.19%), with highest concentration in the three hill districts (11%). Findings revealed superficial knowledge on malaria transmission, prevention and treatment by the respondents. Poverty and level of schooling were found as important determinants of malaria knowledge and practices. Allopathic treatment was uniformly advocated, but the 'know-do' gap became especially evident when in practice majority of the ill persons either did not seek any treatment (31%) or practiced self-treatment (12%). Of those who sought treatment, the majority went to the village doctors and drugstore salespeople (around 40%). Also, there was a delay beyond twenty-four hours in beginning treatment of malaria-like fever in more than half of the instances. In the survey, gender divide in knowledge and health-seeking behaviour was observed disfavouring women. There was also a geographical divide between the high endemic south-eastern area and the low-endemicnorth-eastern area, the former being disadvantaged with respect to different aspects of malaria studied.

**Conclusion:**

The respondents in this study lacked comprehensive knowledge on different aspects of malaria, which was influenced by level of poverty and education. A gender and geographical divide in knowledge was observed disfavouring women and south-eastern area respectively. They preferred allopathic treatment for malaria, although a substantial proportion did not seek any treatment or sought self-treatment for malaria-like fever. Delay in seeking care was common. The implications of these findings for programme development are discussed.

## Background

Malaria is a public health problem in some ninety countries worldwide including Bangladesh and estimated to be responsible directly for about 3,000 deaths a day worldwide [1]. The poor and vulnerable populations are disproportionately affected by malaria and the severe consequences of malaria are borne more by the poorest [2]. There is also strong evidence that the use of preventive and treatment interventions for malaria depends upon socio-economic status (SES) [3-5]. The economic burden of ill health, such as malaria, on individual households can be substantial and in some cases catastrophic, especially for the poor households [6]. Prevention and control of malaria thus can contribute towards poverty alleviation efforts in Bangladesh.

In Bangladesh, the National Malaria Control and Prevention Programme is currently being implemented by the Malaria and Parasitic Disease Control (M&PDC) unit of the Directorate General of Health Services (DGHS), Government of Bangladesh [7,8]. M&PDC implements the programme in the community in partnership with a BRAC-led consortium of 20 small NGOs (selected through competitive bidding) under funding from GFATM round 6 [8]. BRAC, an indigenous micro-credit/micro-finance-based NGO, is working with the twin objectives of alleviation of poverty and empowerment of the poor [9]. As part of its efforts to mitigate the income-erosion consequences of illnesses for the poor households, BRAC is involved in malaria control activities in the three high endemic hill tracts districts since 1998. This current five-year programme (2007–2012) implemented in all of the 13 endemic districts has both preventive (distribution of LLIN/ITNs, intermittent insecticide residual spray and awareness building programmes) and curative (presumptive case management, early diagnosis and prompt treatment following WHO guidelines, and referral of complicated cases to tertiary facilities) components towards reducing malaria morbidity and mortality [7].

A baseline survey was done during the inception phase of the programme to estimate the parasitological prevalence of malaria infection and record benchmark information on the awareness and knowledge of the community regarding the transmission, prevention and treatment of malaria and relevant health-seeking behaviour. The data generated is expected to fill in the knowledge gaps in social science aspects of malaria in Bangladesh and help programme develop informed intervention components and strategies (by BRAC and other NGOs), and also, future programme evaluation and impact assessment [10].

## Methods

**Figure 1:**
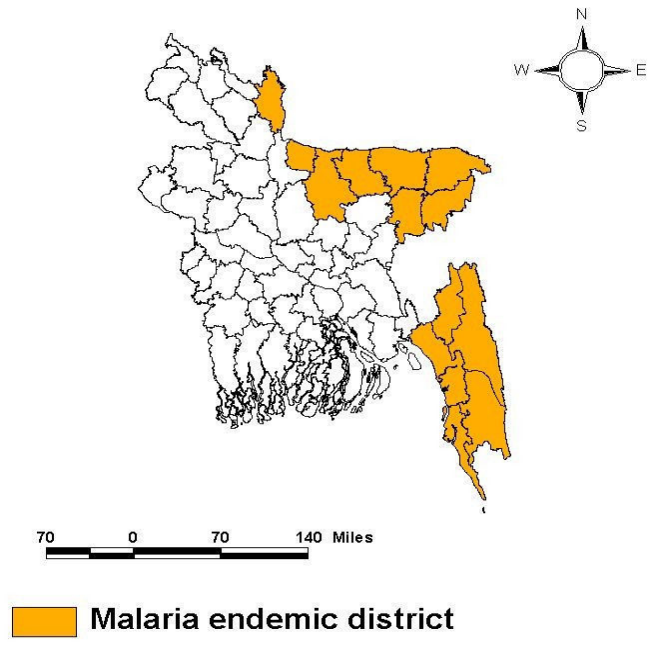
**Two Malaria endemic areas of Bangladesh**.

Data for this paper originated from the baseline survey conducted during July to November 2007, to cover the peak malaria season in Bangladesh. The 13 malaria-endemic districts were divided into two groups based on endemicity [11]: the five high endemic (parasitic prevalence 7.2%) south-eastern districts (henceforth SE area), and the eight low endemic (parasitic prevalence 0.5%) north-eastern districts (henceforth NE area) (Figure 1). The districts are composed of *upazilas *(sub-districts) divided into Unions, the latter again divided into *mauzas *(lowest administrative unit equivalent to, but not necessarily equal to, villages).

### Sampling

Two-stage cluster sampling technique was employed to select the study sample. City Corporations and towns were excluded from this survey. For each of the 13 districts, all *mauzas *were listed and 30 *mauzas *were selected using a probability proportional to size (PPS) sampling procedure [11]. These *mauzas *were the primary sampling unit. Twenty-five households were selected using systematic random sampling from each *mauza*. Sample size was calculated using web-based software (C-Survey 2.0) based on the conservative estimates of malaria prevalence of 2%, design effect of 2, and precision of 1.5% at 95% confidence interval. This yielded a sample size of 750 individuals in each district for the study (total = 9,750).

### The survey

The survey consisted of two parts: parasitological prevalence estimation by RDT and socioeconomic survey related to malarial knowledge and relevant health-seeking behaviour. For the latter, a semi-structured questionnaire was developed and pre-tested for ascertaining consistency, appropriateness of language, and sequencing before finalization [see Additional file 1]. The survey team comprised of experienced social science graduates who received rigorous training for five days on questionnaire content, probing techniques and strategies to establish rapport and neutrality essential to complete and accurate data collection. In hilly areas interviewers from ethnic groups were recruited to interview respective ethnic group of people.

In each *mauza*/village, the survey team selected every third household encountered as they moved from the center or periphery of the *mauza*/village following a designated path using the "spin the bottle" methodology [12]. When there were no respondents present in the selected households or the respondents refused to participate, the teams substituted it with an adjacent house. Beside estimating parasitological prevalence by RDT (Rapid Diagnostic Tests, 'FalciVax' by Zephyr Biomedicals, India) from one randomly chosen member (age>1 year) of each household, the survey questionnaire was administered in a face-to-face interview to the participant if adult or the household head (or spouse or any knowledgeable member of the household) if children to elicit information. Information was also collected for any febrile illness in the past 15 days among the household members and relevant health-seeking behaviour. Patients diagnosed as having malaria at the time of survey were referred for treatment as per national guideline.

The day-to-day field activities of the teams were fine-tuned by field researchers based in local offices and supervised by the principal author. Whenever necessary, re-interview was done by the supervisors for securing reliable and valid data. To improve the latter, an independent quality control team spot-checked households randomly within three days of the main survey. In cases where inconsistencies were noted, interviewers were accompanied by field supervisors until quality standards were met.

### The variables

When individuals living together took meal from a common cooking facility, the entity is defined as a Household (HH). The head of Household is defined as the person who was perceived by household members to be the primary decision-maker in the family and who may or may not have been the main income-earner. Education was measured by completed years of formal schooling. Engagement in a particular income-earning activity for the major part of the day was categorized as 'main occupation'.

Wealth index was constructed following the method developed by Filmer and Pritchett [13]. The assets included for developing the index were: table, bed-cot, quilt, watch, radio, television, bi-cycle and electricity. Each of the variables was recorded into categorical dichotomous (yes, no) variable. Eight dichotomous variables were created and standardized. The principal component analysis was run with all constructed variables with certain criteria. The component score coefficient matrix was multiplied by the standardized variables to produce factor scores which were termed as household wealth score. The wealth scores were further classified into five quintiles, starting from the lowest (1^st ^quintile, poorest) to highest (5^th ^quintile, least poor).

### Ethical considerations

The study received approval by the research review board and the ethical review board of ICDDR, B. Informed consent was obtained from the participants or their guardians before proceeding with the survey activities. Anonymity of the respondents at all stages of data analysis.

## Results

Overall malaria prevalence rate in the 13 endemic districts was found to be 3.1% (*Plasmodium falciparum *2.73%, *Plasmodium vivax *0.16% and mixed infection 0.19%) by the Rapid Diagnostic Test 'FalciVax' (Zephyr Biomedicals, India) and is reported in detail elsewhere [11]. The prevalence was higher in the five south-eastern districts (7.2% as opposed to 0.5% in the north-eastern districts), with highest concentration in the three hill districts (11%).

The socio demographic and household characteristics of the study population are presented in Table 1 by the two study areas. Majority of the respondents (45%) were in their middle age (40–59 years). A greater proportion of respondents from NE districts (55%) compared to SE districts (45%) did not have any formal schooling, with a sex divide disfavouring the females. For males, involvement in farm activities was the most frequent occupation (about 41%), while this was household chores in case of females (around 65%). Less than 7% of the respondents were from female-headed households. Findings revealed that the households from SE area fared better than the other area when stratified in terms of asset quintiles e.g., the proportion of poorest households was 24% in the SE area compared to 18% in the NE area.

**Table 1:** Socio-demographic and household characteristics of the respondents study areas and sex (%)

	South-eastern districts			North-eastern districts		
	
	M	F	All	M	F	All
Sociodemographic characteristics						
						
Age (yrs)						
≤19	0.1	0.8	0.2	0.5	0.2	0.5
20–39	38.9	25.4	38.0	35.5	24.8	34.8
40–59	43.7	53.2	44.4	45.3	54.9	45.9
≥60	17.2	20.6	17.5	18.8	20.1	18.9
Completed years of schooling						
None	42.8	69.4	44.6	53.6	75.2	55.1
1–5	25.7	17.9	25.2	24.9	17.6	24.4
>5	31.6	12.7	30.3	21.5	7.1	20.6
Main occupation						
Self-employment (agri.)	45.8	11.1	43.4	42.7	2.9	40.0
Self-employment (non-agri)	23.0	3.2	21.6	21.9	2.9	20.6
Wage-labour	10.2	9.9	10.2	17.0	8.8	16.5
Service	10.6	4.8	10.2	7.4	2.9	7.1
Domestic chores	0.7	61.5	4.8	1.1	69.9	5.8
Others*	9.7	9.5	9.7	9.8	12.5	10.0
						
Household head						
Male	---	---	93.3	---	---	93.2
Female	---	---	6.7	---	---	6.8
Household asset quintiles						
Poorest	---	---	23.6	---	---	18.0
2^nd^	---	---	21.3	---	---	21.6
3^rd^	---	---	18.1	---	---	18.5
4^th^	---	---	19.7	---	---	20.3
Least poor	---	---	17.4	---	---	21.6

N	3498	252	3750	5548	408	5999

*beggar, unemployed, too old/sick to work etc.

Tables 2, 3 and 4 present malarial awareness of the respondents with respect to sex, educational attainment and household economic status (as proxied by asset quintiles) respectively. In general, the respondents were aware about the cause ('mosquito bite') and presenting symptoms of malarial illness ('fever with shivering') irrespective of sex, and this awareness increased uniformly with years of schooling as well as level of affluence (p < 0.001). However, when they were asked about its mode of transmission, only around 39% in the SE area and 32% in the NE area could respond correctly ('by bite of mosquito which has bitten a malarial patient') (p < 0.001, Table 2), and was also found to be a factor of schooling years and affluence (p < 0.001, Tables 3 and 4 respectively).

**Table 2:** Reported knowledge on malaria by study areas and sex (multiple responses)

	South-eastern districts			North-eastern districts			χ^2 ^significance
		
	M	F	All	M	F	All	
	a	b	c	d	e	f	c vs f

Causes of malaria							
Mosquito bite	90.7	89.2	90.6	94.4	91.9	94.2	p < 0.001
Fly/insect bite	4.8	4.0	4.7	2.0	1.7	2.0	p < 0.001
Not maintaining neat and cleanliness	10.5	7.6	10.3	16.0	5.2	15.9	p < 0.001
Others	3.5	4.2	5.4	3.5	4.2	3.6	p < 0.001

Symptoms of malaria							
Onset of fever with shivering	76.4	72.1	76.1	83.2	81.1	83.0	p < 0.001
Fever at intervals	19.8	22.3	21.0	25.6	27.7	25.8	p < 0.001
Remission of fever with sweating	10.5	7.6	10.3	16.0	15.2	15.9	p < 0.001
Others	18.5	18.7	18.5	10.3	8.6	10.2	p < 0.001

Mode of transmission							
By bite of any mosquito	33.4	36.3	33.5	32.9	36.0	33.1	ns
By bite of mosquito which has bitten a malarial patient	39.2	29.9	38.6	32.5	31.9	32.4	p < 0.001
Don't know	24.4	28.3	24.7	30.6	27.7	30.4	p < 0.001
Other	3.1	3.6	3.1	6.9	7.4	6.9	p < 0.001

Mode of prevention							
Preventing breeding of mossquito	13.3	6.8	12.9	17.6	18.6	17.7	p < 0.001
Using bednet	84.1	83.3	84.0	84.3	80.9	84.1	ns
Using insecticide impregnated bednet	1.0	0.8	1.0	2.1	2.7	2.1	p < 0.001
Using mosquito repellent/coil	16.1	13.1	15.9	19.9	20.8	20.0	p < 0.001
Other	12.3	15.1	12.5	9.4	10.5	9.5	p < 0.001

Mode of treatment							
Allopathic treatment	96.0	96.0	96.0	98.3	97.3	98.2	p < 0.001
Traditional (Herbal/Kabiraji)	1.7	1.2	1.7	1.1	2.0	1.1	p < 0.01
Faith healing	0.5	0.0	0.3	0.3	0.2	0.3	ns
Homeopathic	0.5	0.0	0.3	0.3	0.2	0.3	ns
Other	0.8	1.2	0.8	0.2	0.5	0.2	p < 0.001

Source of information							
Govt. health worker	21.2	23.5	20.7	25.1	23.5	25.0	p < 0.001
NGO health worker	26.8	18.7	26.3	16.7	14.2	16.5	p < 0.001
Radio/TV/Newspaper	14.5	15.5	14.6	15.2	13.0	15.0	ns
Poster/leaflet	2.4	5.6	2.6	12.9	3.2	2.0	p < 0.05
Neighbours/relatives	34.8	43.8	35.4	48.2	27.1	48.8	p < 0.001
Self	16.9	13.9	15.8	14.7	9.6	11.4	p < 0.05
Other	5.7	6.0	5.7	4.2	4.2	4.2	p < 0.001

N	3499	251	3750	5591	408	5999	

**Table 3:** Reported knowledge on malaria by study areas and completed years of schooling (multiple responses)

	South-eastern districts				North-eastern districts			
	
	Completed years of schooling							
	
	None	1–5	>5	χ^2 ^significance	None	1–5	>5	χ^2 ^significance
Causes of malaria								
Mosquito bite	87.4	91.6	94.7	p < 0.001	93.0	95.0	96.6	p < 0.001
Fly/insect bite	4.5	4.5	5.2	ns	1.6	1.9	3.1	p < 0.01
Not maintaining neat and cleanliness	8.3	11.6	12.1	p < 0.001	15.0	13.6	21.2	p < 0.001
Others	7.1	4.5	3.7	p < 0.001	4.4	3.0	1.9	p < 0.001
Symptoms of malaria								
Onset of fever with shivering	71.9	77.2	81.6	p < 0.001	80.0	84.9	88.8	p < 0.001
Fever at intervals	19.2	18.4	22.5	p < 0.05	26.8	22.7	26.7	p < 0.05
Remission of fever with sweating	8.8	11.6	12.1	p < 0.001	15.0	13.6	21.2	p < 0.001
Others	18.5	17.6	19.2	ns	10.9	9.4	9.2	ns
Mode of transmission								
By bite of any mosquito	34.9	37.2	28.4	p < 0.001	33.2	35.7	30.0	p < 0.05
By bite of mosquito which has bitten a malarial patient	29.2	38.9	52.2	p < 0.001	27.5	28.5	49.8	p < 0.001
Don't know	29.2	21.2	20.8	p < 0.001	33.6	31.9	20.2	p < 0.001
Other	3.2	3.1	3.0	ns	7.7	6.3	5.6	p < 0.05
Mode of prevention								
Preventing breeding of mosquito	7.8	12.9	20.3	p < 0.001	16.8	14.0	24.4	p < 0.001
Using bed net	80.9	84.4	88.4	p < 0.001	81.4	85.2	89.7	p < 0.001
Using insecticidal bed net	0.8	1.1	1.3	ns	1.6	2.6	2.9	p < 0.01
Using mosquito repellent/coil	11.2	17.1	22.1	p < 0.001	15.6	21.6	30.0	p < 0.001
Other	14.2	12.4	10.2	p < 0.05	10.9	9.0	6.5	p < 0.001
Mode of treatment								
Allopathic treatment	94.3	96.2	98.3	p < 0.001	97.8	98.3	99.0	p < 0.02
Traditional (Herbal/Kabiraji)	2.0	1.4	1.4	ns	1.0	1.4	1.1	ns
Faith healing	0.5	0.5	0.5	ns	0.2	0.3	0.3	ns
Homeopathic	0.5	0.4	0.3	ns	0.2	0.3	0.6	ns
Other	1.2	0.7	0.4	p < 0.05	0.3	0.1	0.0	ns
Source of information								
Govt. health worker	17.8	19.9	25.5	p < 0.001	22.7	23.5	32.9	p < 0.001
NGO health worker	23.6	25.9	30.6	p < 0.001	15.0	17.0	20.5	p < 0.001
Radio/TV/Newspaper	9.4	16.5	20.7	p < 0.001	12.0	13.4	24.8	p < 0.001
Poster/leaflet	1.5	2.8	4.2	p < 0.001	2.0	1.1	2.9	p < 0.01
Neighbours/relatives	41.6	35.7	25.8	p < 0.001	54.7	48.5	33.2	p < 0.001
Self	13.3	16.6	19.5	p < 0.001	12.0	16.5	18.0	p < 0.001
Other	6.5	6.5	4.0	p < 0.01	4.5	4.5	3.3	ns

N	1683	938	1129		3322	1453	1224	

**Table 4:** Reported knowledge on malaria by study areas and wealth quintiles (multiple responses)

	South-eastern districts				North-eastern districts			
	
	Household asset quintiles							
	
	Poorest	3^rd ^Quin.	Least poor	χ^2 ^significance	Poorest	3^rd ^Quin.	Least poor	χ^2 ^significance
Causes of malaria								
Mosquito bite	84.8	90.7	95.5	p < 0.001	92.2	93.2	96.7	p < 0.001
Fly/insect bite	4.6	5.0	6.5	ns	0.8	1.6	4.4	p < 0.001
Not maintaining neat and cleanliness	7.9	10.6	13.4	p < 0.01	10.1	16.6	22.6	p < 0.001
Others	8.0	5.3	3.4	p < 0.001	5.0	4.3	1.6	p < 0.001
Symptoms of malaria								
Onset of fever with shivering	68.7	76.8	82.9	p < 0.001	76.3	85.0	86.5	p < 0.001
Fever at intervals	17.6	20.6	21.0	ns	25.7	21.7	32.1	p < 0.05
Remission of fever with sweating	7.9	10.6	13.4	p < 0.01	10.1	16.6	22.6	p < 0.001
Others	21.9	17.8	16.1	p < 0.01	12.7	9.0	9.8	p < 0.001
Mode of transmission								
By bite of any mosquito	30.8	33.4	39.3	p < 0.05	29.9	30.1	38.6	p < 0.001
By bite of mosquito which has bitten a malarial patient	30.5	40.1	43.0	p < 0.001	23.7	30.9	43.7	p < 0.001
Don't know	31.0	32.9	18.6	p < 0.001	39.5	32.0	19.3	p < 0.001
Other	1.9	4.0	3.7	p < 0.05	6.4	9.7	4.9	p < 0.001
Mode of prevention								
Preventing breeding of mosquito	6.8	13.1	17.8	P < 0.001	8.9	17.9	26.4	p < 0.001
Using bed net	70.1	84.4	89.4	P < 0.001	84.6	80.4	87.1	p < 0.001
Using insecticidal bed net	0.7	1.5	1.2	ns	0.9	2.7	2.3	p < 0.05
Using mosquito repellent/coil	8.0	15.6	24.6	P < 0.001	13.2	16.4	32.6	p < 0.001
Other	16.9	12.5	8.8	p < 0.001	10.1	11.9	7.0	p < 0.001
Mode of treatment								
Allopathic treatment	92.6	95.7	98.6	p < 0.001	97.2	98.5	98.6	p < 0.05
Traditional (Herbal/Kabiraji)	2.4	1.6	1.7	ns	1.2	1.0	1.2	ns
Faith healing	0.2	0.6	0.6	ns	0.2	0.2	0.4	ns
Homeopathic	0.2	0.1	0.5	ns	0.1	0.2	0.5	ns
Other	1.5	0.6	0.0	p < 0.05	0.6	0.0	0.0	p < 0.001
Source of information								
Govt. health worker	17.8	21.6	22.1	ns	28.1	22.3	26.2	p < 0.05
NGO health worker	23.9	29.7	19.5	p < 0.001	14.3	17.5	16.7	ns
Radio/TV/Newspaper	5.3	13.5	32.4	p < 0.001	4.1	10.5	31.6	p < 0.001
Poster/leaflet	0.9	1.8	6.9	p < 0.001	0.3	2.1	2.7	p < 0.001
Neighbours/relatives	43.9	35.0	28.1	p < 0.001	50.6	53.7	37.9	p < 0.001
Self	12.4	15.0	21.4	p < 0.001	13.3	13.8	18.3	p < 0.001
Other	5.2	6.8	5.4	ns	6.5	3.8	3.6	p < 0.001

N	884	680	651		1082	1108	1294	

Interestingly, the use of bed net for prevention of malaria was singled out uniformly by the respondents (>80%,) (Table 2). Other measures reported were: preventing breeding of mosquito (13% in SE area and 18% in NE area, p < 0.001), using mosquito repellant/coil (16% in SE area and 20% in NE area, p < 0.001). Though there was not much variation by sex (Table 2), but the trend observed earlier with education and affluence remained valid with one exception (Tables 3 and 4 respectively). The respondents almost unanimously reported allopathic medicine to be the treatment for malaria (>98%), especially the more educated and the affluent ones (Tables 3, 4 respectively).

Neighbours and relatives were the most frequently mentioned group for malaria-related information (35% in SE and 49% in NE areas respectively, p < 0.001) by the respondents, especially the females (Table 2). However, with increasing level of education and affluence (p < 0.001, Tables 3 and 4 respectively), the proportion decreased gradually to be replaced by community health workers from government and NGOs. Mass media (Radio/TV/Newspaper) and printing media (poster/leaflet) became increasingly important means of message dissemination in those instances.

Around 2% of the respondents in SE area and 0.4% respondents in NE area reported to have had suffered from fever with shivering within 15 days prior to the day of survey (p < 0.001). No sex difference in fever prevalence was seen (Table 5). Next, information on their health-seeking behaviour was elicited. Majority of these patients did not seek any treatment, women more so than men and those from SE area more so than those in the NE area. Self-treatment was practiced more frequently by patients from NE area (14%) than by those from SE area (11%). Professional allopathic practitioners were consulted in 13% of instances in both areas, with a gender gradient disfavoring women. On the other hand, drugstore salespeople were consulted more frequently by those from the SE area (47%) compared to the NE area (32%), with marginal or no gender difference (Table 5). When disaggregated by education and SES, a decrease in proportion of no-treatment and a simultaneous increase in treatment seeking from professional allopaths i.e., MBBS doctors was observed with increasing level of education and affluence (Table 6).

**Table 5:** Prevalence of fever with shivering in past 15 days prior to the day of survey and relevant health-seeking behaviour %

	South-eastern districts			North-eastern districts			χ^2 ^significance
		
	M	F	All	M	F	All	
	
	a	b	c	d	e	f	C vs f
Had fever with shivering in last 15 days	1.7	2.0	1.8	0.5	0.4	0.4	p < 0.001

N	10147	9675	19822	16025	14962	30987	

Health-seeking behaviour							
No treatment	35.8	41.3	38.7	20.5	25.4	22.7	
Self-treatment	10.4	11.1	10.8	15.1	11.9	13.6	
Drug store salespeople	35.3	29.6	32.3	45.2	49.2	47.0	
Paraprofessionals	2.3	4.8	3.6	2.7	3.4	3.0	
Professional allopaths (MBBS doctors)	14.5	11.6	13.0	16.4	8.5	12.9	
Others	1.7	1.6	1.7	0.0	1.7	0.8	

χ^2 ^significance	ns			ns			

N	173	189	362	73	59	132	

**Table 6:** Health-seeking behaviour of patients by education and affluence %

	South-eastern districts			North-eastern districts		
	
	Years of schooling					
	
	None	1–5	>5	None	1–5	>5
No treatment	39.1	41.1	28.8	20.7	26.3	10.0
Self-treatment	12.8	7.5	15.3	10.3	13.2	25.0
Drug store salespeople	33.8	31.8	32.2	55.2	39.5	35.0
Paraprofessionals	4.5	1.9	5.1	0.0	5.3	10.0
Professional allopaths (MBBS doctors)	9.0	15.9	16.9	12.1	15.8	20.0
Others	0.8	1.9	1.7	1.7	0.0	0.0

χ^2 ^significance	ns			ns		

N	133	107	59	58	38	20

	Household asset quintiles					

	South-eastern districts			North-eastern districts		
	
	Poorest	3^rd ^Quin.	Least poor	Poorest	3^rd ^Quin.	Least poor

No treatment	47.2	30.6	27.6	29.2	28.6	16.0
Self-treatment	10.4	14.5	10.3	16.7	14.3	24.0
Drug store salespeople	26.4	30.6	37.9	37.5	52.4	36.0
Paraprofessionals	3.2	6.5	3.4	0.0	0.0	0.0
Professional allopaths (MBBS doctors)	11.2	12.9	17.2	12.5	4.8	24.0
Others	1.6	4.8	3.4	4.2	0.0	0.0

χ^2 ^significance	ns			ns		

N	125	62	29	24	21	25

Finally, Table 7 shows that there was delay in initiation of treatment beyond 24 hours in majority of the instances, and the illness also prolonged beyond seven days, especially in the NE area (p < 0.01). This resulted in the disruption of income-earning activities beyond five days, more so in the NE area (p < 0.01).

**Table 7:** Time to treatment initiation, duration, disruption of income-earning and illness expenditure by study areas and sex

	South-eastern districts			North-eastern districts			significance
		
	M	F	All	M	F	All	
	
	a	b	c	d	e	f	c vs. f
Treatment initiated %							p < 0.01
Within 24 hours	44.8	41.7	43.2	24.6	27.3	25.7	(χ^2^)
Beyond 24 hours	55.2	58.3	56.8	75.4	72.7	74.3	
							
Duration of illness %							p < 0.01
≤ 3 days	29.1	33.9	31.7	19.6	21.9	20.5	(χ^2^)
4–7 days	50.5	36.4	43.0	28.3	37.5	32.1	
≥ 7 days	20.4	29.7	25.3	52.2	40.6	47.4	
							
Days income-earning was disrupted (mean)	5.0	5.3	5.1	7.0	9.2	7.6	p < 0.003 (t-test)

*beggar, unemployed, too old/sick to work etc.

## Discussion

The role of social science research in the design and implementation of evidence-based prevention, management and control strategies for malaria cannot be overemphasized [14]. There is lack of this kind of data in Bangladesh and this baseline survey on malaria has attempted to fill in this knowledge gap besides estimation of parasitological prevalence from a population based survey in the 13 endemic districts. Findings revealed superficial knowledge on malarial transmission, prevention and treatment, especially among the poor and the illiterate. A gender and geographical divide with respect to different aspects of malaria prevention and treatment was observed, disfavouring women and south-eastern area respectively. While the respondents preferred allopathic providers for treatment of malaria unanimously, a 'know-do' gap in practices existed. In about half of the instances, a delay in seeking care for malaria-like fever was observed. These are discussed below with its implications for programme implementation

The awareness of the respondents that malaria is caused and transmitted by bite of mosquito is usually a common knowledge in malaria endemic countries such as India, Turkey, Nepal, Haiti, Latin America, Sudan and Ghana [15-22]. However, only a tiny fraction of the respondents could accurately state the correct transmission route ('by bite of mosquito which has bitten a malarial patient') and none could state how the mosquito becomes infective i.e., the parasitological cause. The serious gaps in knowledge are also revealed by one-third of the respondents stating that they did not knew the mode of transmission and another one-third stating that any mosquito bite causes malaria. The poor and the semi-literate are especially disadvantaged in these aspects. Health education interventions should be designed according to the existing knowledge and awareness level of vulnerable population as well as their current treatment-seeking practices, and should be implemented for sufficient length of time to be effective [20].

The association of febrile illness with malaria has been known in Bangladesh for a long time [23]. This is also reiterated in this study, where the majority of the respondents mentioned fever (with shivering, at intervals) as the most common symptom of malaria and is consistent with observations from other countries [16,18,20,21,24-26].

Knowledge on the use of bed net as a preventive measure against mosquito bite was high among the respondents in this study. Similar high level of knowledge on preventive use of bed net had been observed in Nepal [18] and Ghana [22], but not in countries such as Ethiopia [25], Iran [26], Delhi, India [16], Turkey [17], and Haiti [19]. This advantage will make the work of the programme easier in introducing insecticidal bed nets (LLINs/ITNs) as a strategic measure for preventing malaria transmission. However, the programme also needs to keep the equity perspective in focus, while distributing insecticidal bed nets, because the poorer households were found to be disadvantaged in this respect.

The respondents were unanimous about seeking treatment from the allopathic providers, whether in the formal or informal sector. However, the 'know-do' gap became especially evident when in practice majority of the ill persons either did not seek any treatment or practiced self-treatment. The latter is consistent with findings from Turkey, where the majority practice self-treatment for malaria [17]. Of those who sought treatment, the majority went to the informal allopathic providers, such as village doctors and drugstore salespeople whose knowledge and capacity for curative treatment is not without question [27]. Also, there was a delay in the beginning of treatment in more than half of the instances of febrile episodes suggestive of malaria, and there was disruption of income-earning activities due to prolonging of the illness. Thus, efforts will be needed to educate this population on the need for 'Early Diagnosis and Prompt Treatment (EDPT)' for reducing its income-erosion effect. Further, the capacity of the informal allopathic providers (important for treatment of poor) should be developed in the use of Rapid Diagnostic Tests (RDTs), and the rational use of artemisinin-based combination drugs (such as Coartem^®^) so as to fast-track informed diagnosis and treatment.

Throughout this study gender divide in knowledge, awareness and health-seeking behaviour was observed disfavouring women. This is not surprising, given the patriarchal norms in the society and was also noted earlier in other studies [28]. While designing interventions, pro-active measures should be undertaken by malarial prevention and control programmes to reduce this gender gap. This is all the more necessary because experiences show that even women-focused interventions may not increase access of quality health care for women, if the gender issues are not explicitly addressed by the programme [29].

Lastly, there are regional differences. The SE area was found to have greater proportion of poorest households (in terms of asset quintiles) than the NE area. The SE area respondents also appeared to be disadvantaged regarding different aspects of malaria prevention and treatment than the NE area, though marginally. However, this difference has to be taken into consideration while allocating resources for specific interventions.

## Conclusion

The findings of the survey have important implications for fine-tuning the current malaria prevention and control programme. The programme should disseminate comprehensive information on different aspects of malaria for converting the 'unfelt' need to 'felt' need of the community to facilitate the uptake of preventive and curative measures. This is all the more necessary as it has been found elsewhere that knowledge of malaria influences the use of preventive measures such as use of insecticidal nets (30). Intensive campaign for practicing EDPT is necessary so that the community is convinced about its need for reducing malaria mortality, especially among the vulnerable groups. Besides print and electronic media, various informal communication methods (e.g., folk songs, people's theatre etc.) can be used to reach the disadvantaged sections of this largely illiterate community. Finally, equity focus in terms of gender, SES and geographical location should be maintained at every stage of programme implementation.

## Competing interests

The authors declare that they have no competing interests.

## Authors' contributions

SMA conceptualized and designed the study, analysed data, drafted the manuscript and made final revisions. RH did sample calculations and designed the study, analysed data and made critical revision of the manuscript. UH organized the field activities, analysed data and helped in the revision of the manuscript. AH analysed data, made the tables and helped in drafting the manuscript. All authors read the final manuscript and approved.

## Supplementary Material

Additional file 1 **Malaria Baseline Survey 2007: Questionnaire**. Questionnaire used in the Baseline Survey. Click here for file
