# Supplementary material for: Knowledge on the transmission, prevention and treatment of malaria among two endemic populations of Bangladesh and their health-seeking behaviour
Source: Malar J. 2009 Jul 29;8:173. doi: 10.1186/1475-2875-8-173 (PMC2729311; doi:10.1186/1475-2875-8-173)
Supplement: Additional file 1 — Malaria Baseline Survey 2007: Questionnaire. Questionnaire used in the Baseline Survey. [file 1475-2875-8-173-S1.pdf]

Malaria Baseline Survey 2007
Questionnaire

Section: I

Household ID No | | | | |

A. Identification:

Id 1.	1= Non NGO member, 2= BRAC member. 3= Others NGO member, 4= Ultra-poor	If necessary please see card/pass book for confirmation					
Id 2.	Households location						
Id 3.	Mauza name (code)						
Id 4.	Union/Pauroshava name						
Id 5.	Upazila name (code)						
Id 6.	District name (code)						
Id 7.	Respondent* name						
Id 8.	Data collector's name						
Id 9.	Date:					'0	7

This questionnaire has been checked properly and no anomaly was observed-

Signature of the supervisor: …………………………….. Date: …………………

* Respondent: Household head/ husband or wife of the household head/ person who knows better about that household

…………………………………………………………………………………………………….

Instruction:

1. Every information obtained form the household should be written in English in this surveillance form. No block of this questionnaire could remain empty.
2. We should obtain present status of the household that you assigned. What will be happening in the future is not the part of this survey.

HOUSEHOLD COMPOSITION

B. Information on Household Members

Sl No		Relation with the household head	Sex M =1 Fe =2	Age (Year/Month)		Marital status	Education	Type educational institute	Main occupation	Physical disable	Feeling sick within last 15 days
				Year	Month						
1	2	3	4	5	6	7	8	9	10	11	12
01		1= household head									
02											
03											
04											
05											
06											
07											
08											
09											
10											
11											

3. Relation with the household head
2= Husband/Wife
3= Son/Daughter
4= Father/Mother
5= Brother/Sister
6= Daughter in law
7= Grandson/ Granddaughter
8= Servant
99= Other (specify)

7. Marital status
1=Unmarried
2=Married
3=Widower/Widow
4= Separated
5= Divorced
88= Not applicable

8. Educational status
00=No formal education
01, 02, ..=Class passed
22=Non graded class
88= Not applicable

9. Type of educational institute
01=Govt. primary
02= Private primary
03=NGO primary
04=Community/ Satellite
05=Ebtedia Madrasha
06= Dakhil Madrasha
07=Alim/Fazil/ Kamil Madrasha
08= Hafizia/Kaumi/ Kharizi Madrasha
09=Kinder Gertain
10= Lowe secondary
11= Private secondary
12=Govt. secondary
13=School/college
14= College/University
77=Don't know
88= Not applicable
99= Other (specify

10. Occupation
01=Farmer (own)
02=Farmer (Barga)
03=Daily labour
04=Service
05=Business
06= Rikswa/Van/Boat Pooler
07=Carpenter/Mason
08= Bus/Tempo/Scooter driver/helper
09= Hotel/Restaurant worker
10=Small business
11= Beggar
12= Working on other's house for food
13=House work
14= Student
15=Unemployed
88= Disable/Not applicable
99= Other (specify

11. Physical disable
1=Blind
2= Deaf
3= Can't talk
4= Can't walk
5= Can't move hand
6=Physically not impaired
99=Other (specify)

12. Sickness
0=No
1=Yes (please fill up the Health Seeking Behaviour Form)

C. Malaria awareness

	Question	Code	Remarks
1.	Have you hared about malaria?	1= Yes; 2= No	
2.	Why some one gets malaria?	1=Mosquito bites; 2=Fly/insect bites; 3=Lack of cleanliness; 4=others (specify)	
3.	How do you know that you have got malaria? (Symptoms of malaria)	1=Fever with rigor; 2= intermittent fever; 3= Fever with sweating; 4=others (specify)	
4.	How malaria is transmitted from one to another?	1= Bitten by any mosquito; 2= Bitten by a mosquito which fed by a malaria patient; 3=others (specify)	
5.	How malaria could be prevented?	1=Limiting mosquito’s breeding places; 2= Bed net; 3= Mosquito coil/ointment; 4= ITN; 5= others (specify)	
6.	How would you treat if you get malaria?	1= Allopathic; 2=Kabiraji/ unani; 3=Spiritual; 4=Homeopathic; 5= others (specify)	
7.	Where you can get this treatment?	1= Govt Hospital; 2= Private health center; 3=Village doctor; 4=Drug seller; 5= others (specify)	
8.	How did you obtain this information?	1= Govt health worker; 2=Non-Govt health worker; 3=Radio; 4=Newspaper; 5=Television; 6=Poster, leaflets etc ; 7= others (specify)	
9.	Is there any BRAC Shastho Sebika (SS) in your area?	1= Yes; 2= No	
10.	If present, did she provide any malaria treatment?	1= Yes; 2= No	
11.	Whom are the others providing malaria treatment in your area?		

D: HEALTH SEEKING BEHAVIOUR FORM FOR HOUSEHOLD MEMBERS WITH MALARIA

Only for those who got code 1 in the column 12 at Household Composition Form

Id no	Name of the sick person	Symptoms (by the sick person or his relatives)	What measure was taken while sick (treatment that was taken first)	If treatment received then how many days later? (nos. 4 to 10 in col. 4)	Treatment cost for last 15 days			How many days was sick (from the beginning till to the end)	Due to illness was any loss of daily wages ? (only for the person aged more than 6 years)	If the answer is for column 8 then how many days?
**1**	**2**	**3**	**4**	**5**	**6**			**7**	**8**	**9**
			No measure was taken [0]<br>Home made traditional medicine/OTC was taken [1]<br>Village doctor [2]<br>Paramedics (MA/FWV/SS/ CHW/HA/ FWA) [3]<br>Drug seller (when diagnosed and given allopathic drugs) [4]<br>Qualified Govt/private MBBS doctor [5]<br>Kabirag/Hekim [6]<br>Spiritual [7]<br>Homeopathic [8]<br>Others specify	1=Within 24 hours<br>2=Within 72 hours<br>3= After 3-7 days<br>4=After 1 week<br>5=Not taken to HCP<br>99= Still sick	Visit (in Taka)	Medicine (in Taka)	Transport cost (in Taka)	1= 3 days<br>2= 4-7 days<br>3= More than 1 week<br>99= Still sick	1= Yes<br>2= No<br>99= Still sick	99= Still sick

*** Health Care provider (HCP): Nos. 2 –8 in column 4**

Section: II

Socio-economic status:

A. Description of household owned land holdings

Description	Own land Own cultivated	Own land Cultivated by Others	Own land Uncultivated (Abundant)	House on own land	Pond portion	Total
Amount of land (decimal)						

Note: During data collection if any one occupying land of his father then the amount of land that he is occupying should considered his own property. If any one cultivated his own mortgaged land then give a note to the field 'Own land Own cultivated'.

B. Household Assets:

Do you have following things in your household?
(use appropriate code for every answer for every question)

Property	Code*	Property	Code*	Property	Code*	Property	Code*	Property	Code*
Electricity		Bed net		Motor cycle		Power tiller		Dheki	
Radio		Rice mill		Crushing mill		Shallow machine		Cattle	
Television		Riksha/Van		Lep/ Mattress		Clock		Goat	
Khat /Choki		Bi-cycle		Chair/Table		Almirah		Fish hatchery	
Net		Duck/Chicken		Variety store (mudi)		Reserved shari		Boat	
Mobile		Others (specify)							

* 1= Yes, 2=No, 3= Shared with Others

C. Status of the living room

Main (largest, well) living room *	Roof	Partition	Floor	Comment
	1= straw/ thatch<br>2=Tin<br>3=Concrete/Cement<br>99= Others (specify)<br>………………….	1= Jut stick/bamboo<br>2= Tin<br>3= Concrete/Cement<br>4= Mud<br>99= Others (specify)<br>………………….	1= Mud<br>2= Cemented<br>3= Semi-cemented<br>99= Others (specify)<br>………………….	1= Jhupri<br>2= Not Jhupri<br>(If there is a permanent structure then it should not be considered as a Jhupri)

* If more than one room present then most expensive and good conditioned room should be selected for collecting information.

D. Perceived HH Economic Status:

(1) What was the household's economic status you feel after observing last one year's income and expenditure from different sources?	All the year deficient…………….1<br>Deficient sometimes …………….2<br>Not deficient nor surplus ………..3<br>Surplus …………………………..4
(2) What kind of economic changes occurred during last one year?	Condition improved ……………..1<br>Not improved…………………….2<br>Condition deteriorated …………..3

Health and hygiene awareness/practices:

E. Self-perception of health

1.	What do you think about your health status at present?	1= Better; 2= Average; 3= Worse
2.	What do you think about your health status at present in compare with previous year?	1= Better than last year; 2= same; 3= Worse than last year

F. General awareness on health and health services:

1.	What should we do to maintain good health? (More than one answer acceptable)	1= Taking nutritious food regularly; 2= Taking bath regularly; 3= Cutting nails regularly; 4= Regular light exercise (eg. Swimming); 5= Keeping clean; 6= All of the above; 88 = Don't know; 99= Others (specify) ……………………………………………….
2.	Do you know about sanitary latrine?	1= Yes, 2= No
3.	What kind of problems may happen if you don't have a sanitary latrine (More than one answer acceptable)	1= Abdominal sickness, 2= Itching, 3= Others (specify) ………………………………………………………..
4.	What are the rules for using sanitary latrine?	1= To wear a sandal; 2=Water pot should be kept on right hand; 3=After deification washing hand with ash and soap; 4= Every age group should use sanitary latrine; 5= It should be cleaned always<br><br>How many answers has given correctly?……….
5.	What kind of latrine do you use?	1=Here and there; 2= Open hole; 3= Canal/river-ponds; 4=Pit; 5= Ring slab; 6= Sanitary ; Others (specify) ………………………
6.	How does water polluted? (Don't prompt; more than one answer acceptable)	1= contact with dirty hands, 2= Contact with dirty and garbage, 3=If lid absent, 9= don't know, 99= Others (specify) ……………………………….. …………
7.	What kinds of diseases are transmitted by polluted water? (Don't prompt; more than one answer acceptable)	1= diarrhea/loose motion; 2= Dysentery; 3= Bloody Dysentery; 4= Indigestion; 5= Worms; 6= Jaundice; 7= Typhoid; 8= Cholera; 10= Arsenicosis; 88= Nothing happen, don't know; Others (specify) ………………………………………………………….
8.	How can we purify water? (Don't prompt; more than one answer acceptable)	1= Boiling ;2= Medicine; 3= Filtering; 88= don't know Others (specify) …………………………………..

9.	What do you do with the domestic garbage that accumulate each day?	1= Certain place (within or outside of the yard); 2=Anywhere; 88= Not applicable, 99= Others (specify) …………………………………………………………
10.	Do you know, if you are sick where you can get treatment in your area?<br>(More than one answer acceptable)	1= Nurse/ Other field level health worker;2=Health and Family welfare center; 3=Upazila health complex; 4= Medical college hospital; 5= District hospital; 6=BRAC health center; 7= Private clinic/ health center; 8= Pharmacy/drug store; 9 MBBS doctor; 10=Kabiraj/Village doctor; 11= Homeopath; 88= Don't know; Others (specify) ……………………………….
11.	If you know, how did you get this information?<br>(More than one answer acceptable)	1= BRAC worker; 2=Knew earlier; 3= Other health worker; 4= Neighbour/friends; 5= Relatives; 6= Radio/TV/Newspaper/Leaflet/ Advertisement at local bazaar; Others (specify) ……………………………….

Thank you for your time and cooperation.
